# Health literacy and breast cancer preventive practices among market women in Oshodi Local Government Area of Lagos State, Nigeria

**DOI:** 10.3332/ecancer.2024.1785

**Published:** 2024-10-09

**Authors:** Omolara Aminat Fatiregun, Yetunde Kuyinu, Adewunmi Alabi, Anthonia Sowunmi, Okunuga Ndidi, Popoola O Abiodun, Temitope Olatunji-Agunbiade, Oyekan Ademola, Nnodimele Onuigbo Atulomah, Awotayo Olajumoke

**Affiliations:** 1 Department of Radiology/Radiotherapy, Lagos State University College of Medicine, Ikeja, Lagos 102101, Nigeria; 2 Institute of Health Policy, Management & Evaluation, Dana Lana School of Public Health, University of Toronto, Toronto, ON M5S 1A1, Canada; 3 Department of Clinical Oncology, Lagos State University Teaching Hospital, Ikeja, Lagos 234001, Nigeria; 4 Department of Community Health and Primary Health Care, Lagos State University College of Medicine, Ikeja, Lagos 102101, Nigeria; 5 Cancer Explore Foundation, Lagos 101245, Nigeria; 6 Department of Radiology, Radiobiology, Radiation Physics, University of Lagos 101017, Lagos State, Nigeria; 7 LUTH/NSIA Radiotherapy Center, Idi-Araba, Lagos 100254, Nigeria PMB 536, Ondo State, Nigeria; 8 School of Public and Allied Health, Babcock University, Ilishan-Remo 121103, Ogun State, Nigeria; 9 University of Medical Sciences, Ondo City, PMB 536, Ondo State, Nigeria; 10 University of Medical Sciences Teaching Hospital, Akure 340283, PMB 536, Ondo State, Nigeria

**Keywords:** health literacy, breast cancer screening, market women, Lagos State

## Abstract

**Background:**

Health literacy connotes understanding health-related issues and applying a clear understanding of implications in making decisions about one’s healthcare needs. Early detection and prompt treatment are cornerstone strategies of breast cancer control. This study assessed the relationship between health literacy and breast cancer prevention practices.

**Methods:**

This study was conducted in Lagos State. Participants’ socio-demographic characteristics, knowledge about breast cancer, attitude towards breast cancer and practice of screening methods available were obtained. Health literacy was assessed with the health literacy domain of a validated questionnaire (Cronbach‘s alpha of 0.75) validated by test-retest reliability) that evaluated the ability to use a language to understand health instructions, cognitive awareness of basic health-related situations, symptom recognitions and health actions required. Health literacy variables were measured on a 19-point rating scale.

**Results:**

Most participants(40%) were between the ages of 31 and 40, while women aged 60 years and above constituted the least proportion (3.1%) of the sample. The mean health literacy score was 12.27 (SD+1.5). A significant proportion(78.4%) of the women had heard of breast cancer. Participants with university/HND education are less likely (OR = 0.431; 95%CI = 0.039,0.759) to have low health literacy. Also, participants with higher income were less likely to have low health literacy, and knowledge of breast cancer risk factors was generally low.

**Conclusion:**

This study shows an above-average mean health literacy score amongst these women; however, inadequate knowledge of risk factors still exists. Education level and income are significant in increasing health literacy on breast cancer preventive practices amongst market women in Lagos, Nigeria.

## Introduction

The challenge of low health literacy among women related to breast cancer prevention can be an essential risk factor for delayed health-seeking behaviour in breast cancer prevention dynamics and predispose such persons to a significant disadvantage in early diagnosis and prompt breast cancer treatment. Functional health literacy determines the perspective of an individual’s health and the ability to use healthcare information to make informed and appropriate health decisions and follow instructions for treatment. Low health literacy levels in some communities contribute to poor health outcomes [1]. Breast cancer is most common among females worldwide, accounting for 25.2% of all new female malignancies. It is also associated with a high level of morbidity and mortality, as it is also the most common cause of cancer-related deaths [2]. In Nigeria, it is also the most common cancer among women [3]. Early detection and prompt treatment are considered important factors contributing to improving breast cancer outcomes and survival and remain the cornerstone of breast cancer control. Breast self-examination (BSE) is recommended to detect palpable breast abnormalities that may predispose to cancer, and screening mammography is recommended to be performed every 1–2 years for women as a preventive strategy for breast cancer [4–6]. Most breast cancer presentations for clinical interventions in Nigeria are in the advanced stages, hence poor prognosis and reduced survival years. This may be due to a lack of awareness and breast cancer preventive practices, mainly contributing to poor health-seeking behaviours on breast cancer. Studies in Nigeria show that health literacy, perceived information needs and preventive health actions are unacceptably low, and this health information deficit is a crucial factor observed in the reported preventive health action [7, 8]. Considerable improvements in breast cancer control in this population may come from fundamental advances in health literacy or tailored approaches to help women with low literacy navigate local healthcare systems [9–11]. This study assesses health literacy scores, knowledge of breast cancer, risk factors and practice of breast cancer prevention among market women in Lagos State.

## Methods

### Study population

This cross-sectional study was conducted among the market women at Kairo market, the largest in Oshodi-Isolo, a Local Government Area within Lagos State, Nigeria, with about 751 stalls over 6 months. The study included market women >18 years old and respondents with signed informed consent. Using the prevalence of 40% from a previous study, the minimum sample size was calculated using the formula for proportions in descriptive studies. With 10% attrition, the minimum number of participants targeted for this study was 406 [12].

### Sampling method

A two-stage sampling technique was employed to recruit participants for the study. Firstly, women in the market were stratified into two mutually exclusive population groups according to sales transactions: Market women inside shops/kiosks and Market women in roadside stands in the market. Secondly, after stratification, systematic random sampling was used (every fourth woman was recruited in the stalls and the roadside stands daily during data collection) to recruit 200 women in shops/kiosks and 200 women in the market in open spaces.

### Data collection instrument

A semi-structured validated questionnaire (Cronbach’s alpha of 0.75) validated by test-retest reliability) was used. The information obtained from participants included socio-demographic characteristics and medical history, including knowledge of breast cancer, attitude towards breast cancer and practice of these screening methods by the respondent. The questionnaire was developed based on peer-reviewed publications on breast cancer screening methods [13]. Health literacy was assessed with the health literacy domain of a validated questionnaire, with six questions to evaluate the ability to use a language to understand health instructions, cognitive awareness of basic health-related situations, symptom recognitions and health actions required. Data was collected with an interviewer-administered questionnaire daily for 3 months. English and Yoruba languages were used for the interview since almost all participants could speak either one of the two languages.

Privacy and confidentiality were ensured by conducting interviews inside the stalls and at the research stand in the market; questionnaires also contained de-identified information.

### Statistical analysis

Data was analysed using the Statistical Product and Service Solution, version 20. The data was summarised using mean and standard deviation for continuous variables and frequencies/percentages for categorical variables. Tests of significance were conducted using chi-square and Fisher exact test. Binary logistic regression was used to determine independent predictors of poor health literacy with *p*-value <0.05. Health literacy variables were measured on a 19-point rating scale, where scores below ten were considered below average. Health literacy scores between 10 and 14 points above average and above 14 points indicated good health literacy [8].

### Ethical considerations

Ethical approval was obtained from the Health Research Ethics Committee of the Lagos State University Teaching Hospital, Ikeja, Lagos State, and the study was approved (REG. NO.NHREC04/04/2008). Also, formal and verbal notification and permission were obtained from the Primary Health Development Agency, Yaba and the Iyaloja of Kairo market, and the local government of Oshodi/Isolo, respectively. Informed consent was also obtained from respondents before data collection. Respondents were also informed that their participation was voluntary and they could decline participation.

## Results

Four hundred and seventeen participants were eligible for the study; 400 consented and completed the questionnaire. This gave a response rate of 98.2%.

### Socio-demographics

The majority of the participants, 161 (40%), were between the ages of 31–40, while women aged 60 years and above constituted the least proportion, 13 (3.1%) of the sample. 220 respondents indicated to be Christians (55%) and 165 were Islamic (41.3%), about 208 (52%) had secondary education, tertiary institution 121(30.3%) as their highest-level education and 71 (17.8%) of the sample were single women. 286 (71.5%) of the sample majority were married and widows were the least represented in the group, at just 10 (2.5%). Most of the participants were Yorubas, at 310 (77.5%), 73 (18.2%) were Igbo and 17 (4.3%) were Hausa (Table 1). Respondents with 1 -2 children comprised of 32%, and 10% had at least five children. Majority of the respondents earned between 18,000 and 100,000 Naira (USD 12 -70 per month) monthly (66.5%), and 0.5% earned above 200,000 Naira monthly (USD 150/month). Most (76.8%) lived in a rented apartment, while 49% resided in a self-contained one (Table 2).

### Knowledge about breast cancer

A significant proportion (78.4%) of the women had heard of breast cancer, while the remaining 21.6% did not. Television was the most common source of information about breast cancer (25.4%), followed by hospitals (20.4%), friends (15.3%), health talks (15.0%), newspapers (9.6%), family (8.9%) and the Internet was the least expected source (5.4%) of breast cancer information. Respondents described breast cancer as a lump in the breast (29.5%), abnormality in breast tissue (18.5%), a common disease in women (14.2%), a spiritual attack (4.3%) and others (1.5%), most did not know what breast cancer was (32%). Participants were able to recognise breast cancer late symptoms such as breast pain (57.5%), breast lumps (22.4%), bloody nipple discharge (17.8%), irregular menstrual cycle (1.5%) and abnormal mammogram findings (0.8%). The majority of the respondents reported chemotherapy as the treatment option for breast cancer (21.2%). Other treatment options identified included removal of the breast (19.2%), radiotherapy (17.0%), removal of lumps (16.2%), prayer (15.4%), none (5.2%), herbal concoctions (3.3%), supplements (2.0%) and others (0.5%). Most respondents did not think breast cancer was contagious (82.7%), while 5.3% thought it was contagious, and 12.0% were unsure if it was.

Regarding breast cancer risk factors, 27.2% of the respondents considered increasing age a risk factor, 33.8% did not and 39.0% were unsure. A family history of breast cancer was considered a risk factor by 48.0%, while 19.2% did not consider it a risk factor, and 32.8% were unsure. A family history of breast cancer was a risk factor for 49.4% of the respondents, while 20.7% did not see it as a risk factor, and others, 29.9%, were unsure. Oral contraceptives were a breast cancer risk factor by 23.7% of the respondents, whereas 26.3% did not consider it a risk factor, and 50.0% were unsure. Some respondents identified breastfeeding as a risk factor (15.0%), 51.2% did not and 33.8% were unsure. 42% of the participants considered benign breast conditions a risk factor, 10.8% did not consider it a risk factor, and 47.2% were unsure. Alcohol intake was a risk factor for breast cancer by 25.0% of the respondents, while 41.7% did not see it as a risk factor, and 33.3% were unsure. Respondents also indicated obesity as a risk factor for breast cancer (19.5%), but some did not (43.8%), and some were not sure (36.8%) if obesity was a risk factor for breast cancer (Table 3).

### Perception and attitude towards breast cancer

The majority of the women did not consider breast cancer as a death sentence (63.5%), while 16.2% felt it was a death sentence, and 20.3% were unsure. Respondents thought early presentation could improve the chance of a cure (82.5%), 5% thought it would not and 12.5% were uncertain. Most respondents felt it was necessary to have a screening (91.0%), while 4% did not agree, and 5% were unsure. Respondents indicated that the cost of screening was expensive (35.4%), whereas some did not (33.1%), and 31.5% were not sure. The majority of the respondents did not think they could be infected by breast cancer (86.0%). Respondents who indicated that breast cancer could be cured were in the majority (63.4%), while 10.8% indicated that breast cancer could not be cured. Some of the respondents (21.7%) thought that breast cancer was a form of spiritual attack, while the majority (58.0%) did not know it was a spiritual attack (Table 4).

The majority of the respondents thought breast cancer could be prevented (62.5%), 10.2% thought it could not be prevented, and 27.3% were not sure it could be prevented. Respondents who indicated breast cancer could be prevented reported the following as methods of prevention: monthly self-breast examination (36%), clinical breast examination (CBE) (28%), mammography (9.2%), breast ultrasound (6.5%), magnetic resonance imaging (MRI) (2.5%) and others (0.3%). However, 17.5% of these respondents did not know how breast cancer could be prevented.

### Breast cancer prevention practices

56.7% of respondents reported performing self-breast examinations within the last year. In addition, respondents had also performed CBE (22.0%), mammography (12.1%), breast ultrasound (4.1%) and MRI (5.1%) within the last year (Table 5).

### Health literacy

**Figure 1.**
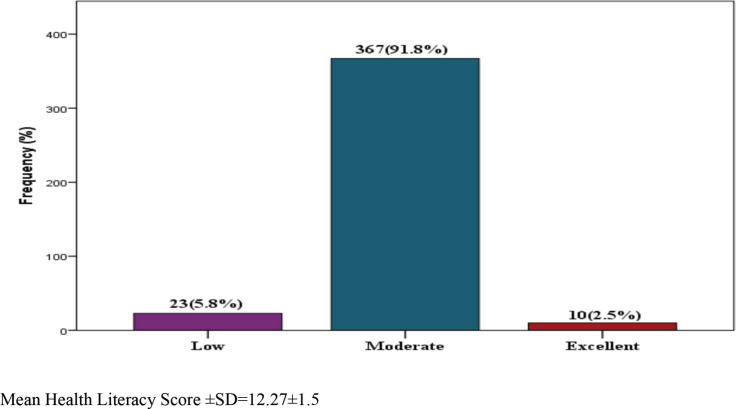
Level of health literacy among participants. Mean health literacy score ± SD = 12.27 ± 1.5.

The mean health literacy score was 12.27 (SD+1.5). Most participants, 367 (91.8%), have moderate health literacy, while only 10 (2.5%) have excellent health literacy (Figure 1). Age group, marital status, level of education and estimated income were associated with different levels of health literacy (*p* < 0.05). Participants above 60 years old, that had marital issues, low education, and less income were more likely to have a low level of health literacy. Binary logistic regression shows the level of education and estimated monthly income as predictors of low health literacy. Participants with university/HND education are less likely (OR = 0.431; 95% CI = 0.039–0.759) to have low health literacy. Also, participants with an estimated monthly income above 18,000 nairas are less likely to have low health literacy (Table 6).

## Discussion

Breast cancer is a disease of high burden in Nigeria. Health literacy is an important cognitive resource associated with women’s health-seeking behaviour, including breast cancer prevention strategies [14]. In this study, Lagos residents practice different religions; Christianity and Islam are the two predominant religions, and respondents were primarily Christians. Most respondents had at least a secondary school education level because of the Lagos state’s free education program [15]. Most of the women in this study were married. Another study conducted among market women in Ibadan, Nigeria, shows such a distribution. It was also found that most respondents were between 30 and 49 years old and were married [16], and most had 3 to 4 children.

Most respondents earned between 18,000 and 100,000 Naira (USD12-70) monthly, which may not be sufficient to afford them the proper screening test required for early breast cancer detection and management. This is also below the national minimum wage of 30,000 Naira [17]. This study reported satisfactory awareness of breast cancer among the respondents. This may be attributed to the free breast cancer screening campaigns frequently organised by the Lagos state government [18] and other non-government organisations (NGOs) [19, 20]. More than 80% of the respondents had at least a secondary level of education. This finding is similar to a study conducted in other parts of the country [21, 22].

In this study, some respondents did not know what breast cancer was. However, respondents in this study were able to identify the signs of breast cancer, contrary to findings in another study done among women in Edo state, where participants’ knowledge about breast cancer symptoms was poor [13]. Many respondents were knowledgeable about the risk factors for breast cancer. Another study done among female healthcare workers in southern Nigeria had similar findings to this present study, and more than half of the participants knew that a positive family history of breast cancer is a risk factor for the development of breast cancer, followed by the use of contraceptive agents [23]. However, as seen in the present study, the knowledge of breast cancer risk factors might be related to the educational level of the respondents, as more than three-quarters of the population had at least a secondary level of education.

Regarding respondents’ perceptions and attitudes towards breast cancer prevention, most respondents believed that breast cancer was not an automatic death sentence, and that early presentation could lead to a potential cure and that the screening was necessary. Still, only a few believed screening was expensive. Other studies also found that respondents indicated that breast cancer could be cured if detected early [21, 24]. The majority of the respondents also believed that breast cancer could be cured, while a few thought breast cancer was a spiritual attack. However, contrary to the findings of this present study, it was noted in a study done among the female academic and non-academic staff of fifteen faculties of the University of Nigeria, Nsukka, Enugu state, that the majority of the sample population believed that breast cancer could not be cured [24]. Another study, in agreement with this present study, noted that only a few respondents believed breast cancer was a spiritual attack [22].

Concerning the practice of breast cancer prevention, more than half of the respondents in this study thought that breast cancer was preventable, and a third of the respondents indicated BSE and CBE as preventive methods, while very few selected mammography, breast ultrasound and MRI. Another study, similar to the present study, showed that only a third of the respondents indicated BSE to prevent breast cancer [22]. However, contrary to these, another study among women in Ibadan found that greater than two-thirds of the sample population indicated BSE was a prevention method for breast cancer [16]. In addition, a few respondents also stated in another study that mammography prevents breast cancer [13].

Over half of the participants indicated that they had done a self-breast examination within the last year. Still, only a few had done CBE, mammography, breast ultrasound or MRI within the previous year. This may be related to the respondents’ good education level, and some studies showed that good education is linked to better breast cancer prevention strategies [25]. In another study, the practice of BSE, CBE and mammography was lower as most respondents had not practised breast cancer prevention [21]. Another study also found that only a third of the study population had performed BSE; one out of ten had performed CBE, and none had mammography [13].

The general health literacy of respondents was above average; this study reported a marginally excellent overall health literacy score. The high health literacy in this present study could be because most respondents had at least a secondary level of education. However, some domains of their health literacy were particularly low, similar to that reported in other studies [8]. The study shows that the level of education and estimated monthly income are independent predictors of low health literacy. University/HND education participants are less likely to have low health literacy. Also, participants with an estimated income above 18,000 nairas are less likely to have low health literacy. Income levels and education are significant in health literacy and breast cancer preventive practices.

## Limitations

A significant limitation of cross-sectional studies, such as this study, is its inability to establish causation. It could only establish an association between health literacy and breast cancer preventive practices. In addition, the study was also susceptible to recall bias, as participants might have difficulty remembering some of the details of questions asked in the questionnaire. The study might have excluded participants with a breast cancer patient in the family and or among relatives. Furthermore, the study participants are from Lagos state’s Oshodi market, an urban/peri-urban site with a better level of education compared to rural areas of Nigeria; this might impact the generalizability of the study.

## Conclusion

This study has shown an above-average health literacy score on breast cancer preventive practices among women in Lagos state’s Oshodi market. However, inadequate knowledge of its risk factors still exists. Income levels and education are significant factors in improving health literacy and practices of breast cancer prevention amongst market women. These are targetable and implementable findings that can be incorporated into health policy development and health campaigns as a continued effort to improve knowledge and uptake breast cancer preventative practices in Lagos State. As well as, an inclusion of either free or subsidised breast screening modalities in order to increase breast cancer prevention practices among women in Lagos State.

## Conflicts of interest

The authors declare no conflicts of interest.

## Funding

No funding was received to conduct the study.

## Figures and Tables

**Table 1.** Socio-demographic characteristics of the respondents.

	Frequency (*N* = 400)	Percentage (%)
Age (years)		
≤20	13	3.3
21–30	68	17.0
31–40	161	40.3
41–50	105	26.3
51–60	40	10.0
>60	13	3.1
Religion		
Christianity	220	55.0
Islam	165	41.3
Traditional	7	1.7
Others	8	2.0
Marital status		
Single	71	17.8
Married	286	71.5
Divorced	16	4.0
Separated	17	4.3
Widow	10	2.5
Level of education		
None	7	1.8
Primary	60	15.0
Secondary	208	52.0
University/HND	121	30.3
Ethnicity		
Yoruba	310	77.5
Igbo	73	18.2
Hausa	17	4.3

**Table 2.** Socio-economic characteristics of respondents.

Variables	Frequency (*N* = 400)	Percentage(%)
Estimated monthly income		
<18,000 (Naira)	78	19.5
>18,000-<100,000 (Naira)	266	66.5
>100,000- <200,000(Naira)	54	13.5
>200,000 (Naira)	3	0.5
Housing type		
One room	74	18.5
Two-room self-contained	196	49.0
Flat	112	28.0
Duplex	11	2.8
Others	7	1.7
Housing status		
Rented	307	76.8
Inherited or self-owned	93	23.2

**Table 3.** Respondents’ knowledge of risk factors for breast cancer.

	Yes	No	Not sure
Risk factors			
Increasing age	86 (27.2)	106 (33.8)	122 (39.0)
Family history	151 (48.0)	60 (19.2)	103 (32.8)
Personal history of breast cancer	155 (49.4)	65 (20.7)	94 (29.9)
Oral contraceptives	75 (23.7)	82 (26.3)	157 (50.0)
Breastfeeding	47 (15.0)	161 (51.2)	106 (33.8)
Benign breast conditions like lumps	132 (42.0)	34 (10.8)	148 (47.2)
Alcohol intake	79 (25.1)	131 (41.7)	104 (33.2)
Obesity	61 (19.4)	138 (43.9)	115 (36.7)

**Table 4.** Perception attitude towards breast cancer preventive practices.

	Yes	No	Not sure
Breast cancer is a death sentence	51 (16.2)	199 (63.5)	64 (20.3)
Early presentation can improve cure	259 (82.5)	16 (5.0)	39 (12.5)
It is necessary to have screening	286 (91.0)	13 (4.0)	15 (5.0)
Screening is expensive	111(35.4)	104 (33.1)	99 (31.5)
Breast cancer can infect you	9 (2.7)	270 (86.0)	35 (11.3)
Breast cancer can be cured	199 (63.4)	34 (10.8)	81 (25.8)
A spiritual attack can cause breast cancer	68 (21.7)	182 (58.0)	64 (20.3)

**Table 5.** Practice of breast cancer prevention.

	Frequency (*N* = 314)	Percentage
Which screening have you undergone in one year?		
Self-breast examination	178	56.7
Examination by Doctor	69	22.0
Mammography	38	12.1
Breast ultrasound	13	4.1
MRI	16	5.1

**Table 6.** Logistic regression showing independent predictors of low health literacy.

	Odd ratio	95% CI	*p*-value
Age group (Years) ≤40 >40	1 1.506	0.498–4.551	0.468
Marital status Single Married Others	1 1.478 4.437	0.269–8.133 0.606–32.493	0.653 0.142
Level of education None Primary Secondary University/HND	1 1.217 0.583 0.431	0.203–7.308 0.109–3.126 0.039–0.759	0.830 0.529 0.032*
Estimated monthly income Less than 18,000 Eighteen-hundred thousand Above Hundred	1 0.131 0.212	0.043–0.400 0.186–0.729	<0.001* <0.001*
